# Discovery of a novel allosteric inhibitor-binding site in ERK5: comparison with the canonical kinase hinge ATP-binding site

**DOI:** 10.1107/S2059798316004502

**Published:** 2016-04-26

**Authors:** Hongming Chen, Julie Tucker, Xiaotao Wang, Paul R. Gavine, Chris Phillips, Martin A. Augustin, Patrick Schreiner, Stefan Steinbacher, Marian Preston, Derek Ogg

**Affiliations:** a Chemistry Innovation Centre, Discovery Sciences, AstraZeneca R&D Mölndal, 431 83 Mölndal, Sweden; b Structure and Biophysics, Discovery Sciences, AstraZeneca R&D Alderley Park, Macclesfield SK10 4TG, England; c Innovation Centre China, AstraZeneca Asia and Emerging Markets iMed, Shanghai 201203, People’s Republic of China; d Proteros biostructures GmbH, Bunsenstrasse 7a, 82152 Martinsried, Germany; e Screening Sciences, Discovery Sciences, AstraZeneca R&D Alderley Park, Macclesfield SK10 4TG, England

**Keywords:** ERK5 kinase, allosteric kinase inhibitor, MAPK7 kinase, crystal structure

## Abstract

MAP kinases act as an integration point for multiple biochemical signals and are involved in a wide variety of cellular processes such as proliferation, differentiation, regulation of transcription and development. As a member of the MAP kinase family, ERK5 (MAPK7) is involved in the downstream signalling pathways of various cell-surface receptors, including receptor tyrosine kinases and G protein-coupled receptors. In the current study, five structures of the ERK5 kinase domain co-crystallized with ERK5 inhibitors are reported. Interestingly, three of the compounds bind at a novel allosteric binding site in ERK5, while the other two bind at the typical ATP-binding site. Binding of inhibitors at the allosteric site is accompanied by displacement of the P-loop into the ATP-binding site and is shown to be ATP-competitive in an enzymatic assay of ERK5 kinase activity. Kinase selectivity data show that the most potent allosteric inhibitor exhibits superior kinase selectivity compared with the two inhibitors that bind at the canonical ATP-binding site. An analysis of these structures and comparison with both a previously published ERK5–inhibitor complex structure (PDB entry 4b99) and the structures of three other kinases (CDK2, ITK and MEK) in complex with allosteric inhibitors are presented.

## Abstract 2

Crystal structures of the MAP kinase ERK5 in complex with XMD8-92 and four novel inhibitors reveal an allosteric binding site between the kinase P-loop and αC helix. Binding at this site displaces the P-loop into the ATP-binding site and was shown to be ATP-competitive.

## 1. Introduction   

Mitogen-activated protein kinase (MAPK) signalling pathways are highly evolutionarily conserved throughout eukaryotes and perform key roles in mediating intracellular signal transduction. Of the four mammalian MAP kinases, ERK (extracellular signal-regulated kinase) 1/2, JNK, p38 and ERK5 (MAPK7) (Raman *et al.*, 2007 ▸; Chang & Karin, 2001 ▸; Pearson *et al.*, 2001 ▸), ERK5 is the most structurally divergent of the family and is relatively poorly understood. The ERK5 protein, which is encoded by the *MAPK7* gene (Zhou *et al.*, 1995 ▸), comprises an N-terminal kinase domain and a large C-terminal domain containing transcriptional transactivating functionality and nuclear localization and export sequences (NLS/NES). ERK5 is the effector kinase of a canonical kinase module comprising MEK (MAPK/ERK kinase) 5, MEKK (MEK kinase) 2/3 and ERK5 itself (Lee *et al.*, 1995 ▸).

Under normal physiological conditions, MEK5 and ERK5 are activated by growth factors and cellular stresses (Abe *et al.*, 1996 ▸; Kamakura *et al.*, 1999 ▸) and, through the use of embryonic gene knockouts of *MEK5* or *MAPK7*, have been shown to contribute largely to blood-vessel and cardiac formation during development (Wang *et al.*, 2005 ▸; Regan *et al.*, 2002 ▸). *In vitro* muscle-differentiation systems have highlighted prominent roles for ERK5 signalling in muscle development (Dinev *et al.*, 2001 ▸), whilst in adult tissues the pathway plays a role in regulating the proliferation and survival of endothelial cells and various immune-derived cell populations (Hayashi *et al.*, 2004 ▸; Sohn *et al.*, 2008 ▸; Rovida *et al.*, 2008 ▸; Garaude *et al.*, 2006 ▸).

In the context of cancer, clinical evidence suggests a role for dysregulated MEK5/ERK5 signalling as a driver of tumourigenesis in several disease settings. Specifically, in breast cancer, increased ERK5 protein levels are associated with decreased disease-free survival. Furthermore, MEK5 expression is up-regulated by constitutive activation of STAT3 (signal transducer and activator of transcription 3), which is commonly detected in advanced breast cancer (Song *et al.*, 2004 ▸; Montero *et al.*, 2009 ▸). The ERK5 pathway also appears to play a role in mediating chemoresistance and contributes to neuregulin signalling in breast-cancer cells overexpressing ErbB2 (Weldon *et al.*, 2002 ▸; Esparís-Ogando *et al.*, 2002 ▸). In prostate cancer, MEK5 is overexpressed and is associated with bone metastases, invasive potential and corresponding poor survival (Mehta *et al.*, 2003 ▸). Moreover, in hepatocellular carcinoma (HCC), genetic dysregulation of *MAPK7* expression through amplification of 17p11 is detectable in around 50% of primary HCC tumours (Zen *et al.*, 2009 ▸). In the same study, preclinical validation work using small interfering RNA (siRNA) suppression of *MAPK7* expression in amplified cell lines confirmed a role for dysregulated MAPK7 in controlling mitotic entry. Finally, recent findings from our own laboratories have implicated amplification of *MAPK7* as a potential tumour driver in sporadic cases of oesophageal and lung squamous-cell carcinoma (Gavine *et al.*, 2015 ▸).

Therapeutic intervention through the inhibition of ERK5 kinase activity may thus have utility in oncology, and a small-molecule inhibitor of ERK5 with anti-proliferative effects in both *in vitro* and *in vivo* models of cancer has been reported (Yang *et al.*, 2010 ▸). Several compounds showing ERK5 inhibition in biochemical and cell-based assays have also been described (Tatake *et al.*, 2008 ▸; Deng *et al.*, 2013 ▸). In the work presented here, we describe several novel small-molecule ERK5 inhibitors which were identified as part of a high-throughput screen of our corporate collection. Interestingly, co-crystal structures of these inhibitors with the kinase domain of ERK5 showed that one class of these ERK5 inhibitors comprises novel allosteric kinase inhibitors that bind at an induced-fit pocket adjacent to the ATP-binding site formed as a result of remodelling of the P-loop. The other class, meanwhile, binds at the conventional ATP-binding site. The most potent allosteric inhibitor demonstrates a half-maximal inhibitory concentration (IC_50_) of 2.3 µ*M* in our enzymatic assay, and its ERK5 inhibition is ATP-competitive. The co-crystal structures of our novel allosteric inhibitors are described and compared with those of conventional ERK5 inhibitors and with known allo­steric inhibitors of cyclin-dependent kinase 2 (CDK2), MAPK kinase (MEK) and interleukin 2-inducible T-cell kinase (ITK).

## 2. Experimental procedures   

### 2.1. Cloning, expression and purification   

Human ERK5 (amino acids 46–402) was amplified from synthetic DNA (Life Technologies) and fused to a DNA sequence coding for glutathione *S*-transferase (GST) and a *Tobacco etch virus* (TEV) protease cleavage site (sequence details are provided in the Supporting Information). The resulting construct was cloned into the vector pFastBac HT A using standard molecular-biology protocols, and recombinant baculovirus was produced following the instructions given by the supplier. The protein was expressed in Sf9 insect cells grown in single-use WAVE bio­reactors using a titreless infection protocol at 299 K for 64 h. The cells were harvested by centrifugation, washed with 1× phosphate-buffered saline (PBS) and stored at 193 K until purification.

For purification, frozen cells were thawed in 1× PBS supplemented with 10% glycerol, 5 m*M* dithiothreitol (DTT), cOmplete Protease Inhibitor Cocktail (Roche) and DNase, and were lysed with an Ultra-Turrax. After centrifugation (all purification steps were performed at 277 K), the supernatant was applied onto a 20 ml column of glutathione (GSH) Sepharose (GE Healthcare) and the bound protein was eluted with 10 m*M* reduced GSH. The fusion tag was removed by digestion with recombinant TEV protease overnight whilst dialysing against approximately 100 volumes of buffer without glutathione. Cleaved ERK5 protein was further purified by a second passage over the GSH Sepharose column followed by size-exclusion chromatography on a Superdex 75 26/60 column (GE Healthcare) equilibrated in 20 m*M* Tris–HCl pH 8.0, 250 m*M* NaCl, 10% glycerol, 2 m*M* DTT. ERK5-containing fractions were diluted fivefold with 50 m*M* HEPES pH 6.5, 10% glycerol, 2 m*M* DTT and applied onto a 6 ml Resource S column equilibrated in the same buffer. Protein bound to the column was eluted with a gradient to 200 m*M* NaCl, and ERK5-containing fractions were pooled and concentrated to >12 mg ml^−1^ as determined by a standard Bradford assay.

### 2.2. Crystallization and data collection   

**Figure 1:**
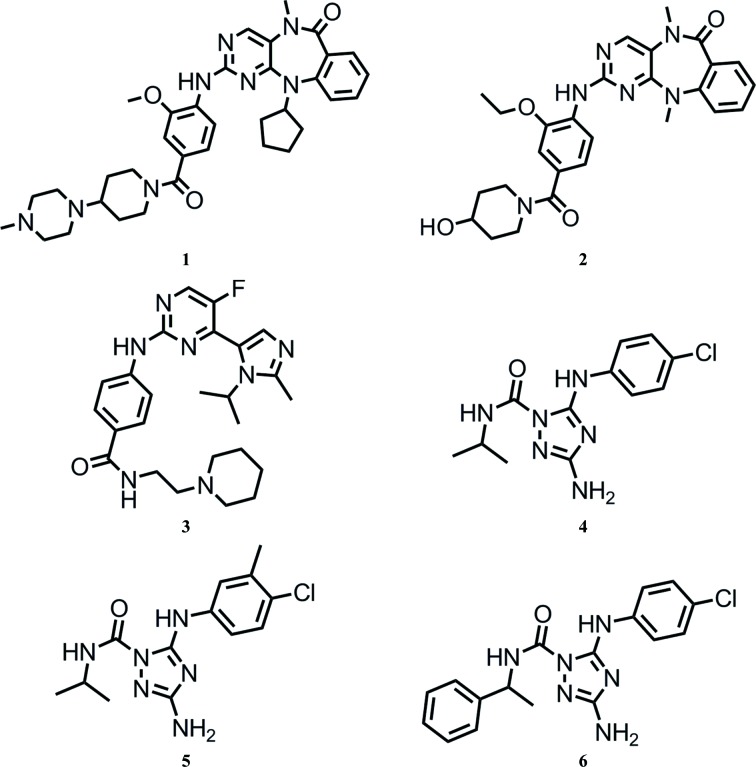
Chemical structures of the ERK5 inhibitors used in this study.

The structure of ERK5 in complex with compound **2** (Fig. 1 ▸) was obtained by soaking the compound at a final concentration of 5 m*M* and 5%(*v*/*v*) DMSO into pre-formed apo crystals [grown from 13%(*w*/*v*) PEG 4000, 180 m*M* sodium formate, 100 m*M* MES pH 6.5, 10 m*M* Tris–HCl pH 8.5, 10 m*M* MgCl_2_] for 30 min at 277 K.

The structures of ERK5 in complex with compounds **3**, **4**, **5** and **6** were obtained by co-crystallization. Purified recombinant human ERK5 kinase domain in storage buffer [50 m*M* HEPES pH 6.5, 120 m*M* NaCl, 10%(*v*/*v*) glycerol, 2 m*M* DTT] was incubated for 3 h on ice with compound diluted from either a 100 m*M* stock in DMSO to a final concentration of 1 m*M* compound, 1%(*v*/*v*) DMSO, 12 mg ml^−1^ protein (compounds **3** and **4**; Fig. 1 ▸) or diluted from a 20 m*M* stock in 2,3-butanediol to a final concentration of 0.2 m*M* compound, 1%(*v*/*v*) 2,3-butanediol, 12 mg ml^−1^ protein (compounds **5** and **6**; Fig. 1 ▸). Samples were then clarified by centrifugation (5 min, 13 000*g*, 277 K). Crystals with compound **3** were grown by hanging-drop vapour diffusion at 277 K by mixing the protein–compound complex with mother liquor [11%(*w*/*v*) PEG 4000, 180 m*M* sodium formate, 100 m*M* MES pH 6.5, 10 m*M* Tris–HCl pH 8.5, 10 m*M* MgCl_2_] in a 0.75:0.5 ratio to give a 2.0 µl drop. Crystals of ERK5 with compounds **4**, **5** and **6** were grown by hanging-drop vapour diffusion at 293 K from a mother liquor consisting of 4–6%(*w*/*v*) PEG 6000, 100 m*M* MES pH 6.25, 5 m*M* DTT using a 1:1 drop ratio and a final drop size of 0.7–1.0 µl. Drops were immediately streak-seeded using a seed stock supplied by Proteros biostructures GmbH. Crystals appeared within 4 d and were harvested after 6 d into a cryoprotectant solution consisting of 70%(*v*/*v*) mother liquor, 30%(*v*/*v*) glycerol. After around 10 s of equilibration, crystals were flash-cooled in a stream of gaseous nitrogen at 100 K.

X-ray diffraction data were collected at the Swiss Light Source (SLS), the European Synchrotron Radiation Facility (ESRF), Diamond Light Source (DLS) and the Australian Synchrotron (AS) (McPhillips *et al.*, 2002 ▸; see Table 1 ▸ for details). Data were integrated, scaled and merged using *XDS* (Kabsch, 2010 ▸) and *AIMLESS* (Winn *et al.*, 2011 ▸; Evans & Murshudov, 2013 ▸). Structures were solved, initial refinement was carried out and the ligand was fitted to difference electron density using the programs *Phaser* (McCoy *et al.*, 2007 ▸), *BUSTER* (Bricogne *et al.*, 2011 ▸) and *rhofit* (Womack *et al.*, 2010 ▸) as implemented in the automated structure-solution pipeline *Pipedream* (Sharff *et al.*, 2011 ▸). A high-resolution structure of the ERK5 kinase domain supplied by Proteros biostructures GmbH was used as the search model for molecular replacement. Coordinates and stereochemical restraints for compounds were generated from the appropriate SMILES strings using *grade* (Smart *et al.*, 2011 ▸). Manual model completion was carried out using *Coot* (Emsley *et al.*, 2010 ▸), interspersed with rounds of refinement in *BUSTER* (Bricogne *et al.*, 2011 ▸). The quality of the final model was verified using *MolProbity* (Chen *et al.*, 2010 ▸), *PROCHECK* (Laskowski *et al.*, 1993 ▸), *Mogul* (Bruno *et al.*, 2004 ▸) and the validation tools available through *Coot* (Emsley *et al.*, 2010 ▸). Full statistics of the data collection, refinement and final model quality are provided in Table 1 ▸. Coordinates, structure factors and full experimental conditions have been deposited with the Protein Data Bank (PDB) under accession codes 5byy, 5byz, 4zsg, 4zsj, and 4zsl for the structures of the complexes of ERK5 with **2**, **3**, **4**, **5** and **6**, respectively. *CCP*4*mg* (McNicholas *et al.*, 2011 ▸) was used for structure alignment and figure preparation. Simulated-annealing OMIT maps were calculated using *phenix.refine* (Afonine *et al.*, 2012 ▸).

### 2.3. Biochemical assay for ERK5 IC_50_ determination   

Catalytically active kinase domain of recombinant human ERK5 enzyme was purchased from Carna Biosciences. The crystallography-grade protein described in §[Sec sec2.1]2.1 is not phosphorylated and thus is inactive and unsuitable for use in the enzymatic assay. The inhibitory potency of compounds against ERK5 was evaluated using the LANCE *Ultra* technology from PerkinElmer according to the manufacturer’s instructions. Briefly, 3 µl of different concentrations of ATP and 100 n*M* U*Light*-eIF4E-binding protein 1 (Thr37/46) peptide (Perkin­Elmer catalogue No. TRF0128) were incubated in the presence of serially diluted compound or DMSO control at room temperature in 384-well Greiner white polystyrene assay plates. The reaction was initiated by the addition of 3 µl of 10 n*M* kinase. The assay buffer consisted of 1 m*M* DTT, 10 m*M* MgCl_2_, 1 m*M* EGTA, 0.01%(*v*/*v*) Tween 20 in 50 m*M* HEPES pH 7.5. After 60 min incubation, the reaction was stopped by the addition of 6 µl stop/detection reagent mixture consisting of 20 m*M* EDTA and 4 n*M* Eu-anti-phospho-eIF4E-binding protein 1 (Thr37/46) antibody (PerkinElmer catalogue No. TRF0216) diluted in detection buffer. The plates were incubated for 1 h before the fluorescence was measured at 615 and 665 nm with an excitation wavelength of 320 nm using an EnVision Multilabel Reader from Perkin­Elmer in time-resolved fluorescence resonance energy transfer (TR-FRET) mode. The calculated signal ratio at 665/615 nm is proportional to the kinase activity. The concentration of compound producing 50% inhibition of the kinase activity (IC_50_) was calculated by using *GraphPad Prism* to fit the data to a symmetrical sigmoidal curve.

### 2.4. Kinase-panel activity screen   

Compounds **2**, **3** and **5** were tested for activity against the 225 kinases in the ThermoFisher standard kinase panel. The standard Z′-LYTE assay protocol (ThermoFisher) was used to measure the percentage inhibition at 10 µ*M* compound concentration (details are provided in the supporting information).

## 3. Results and discussion   

### 3.1. Identification of novel ERK5 inhibitors   

The previously reported ERK5 inhibitor XMD8-92 (compound **2**; Fig. 1 ▸; Yang *et al.*, 2010 ▸) was used as a reference compound in the development and validation of a TR-FRET-based biochemical assay of ERK5 kinase activity (Table 2 ▸). The crystal structure of compound **1** (Fig. 1 ▸), a close analogue of compound **2**, in complex with ERK5 (PDB entry 4b99) has recently been published (Elkins *et al.*, 2013 ▸). The IC_50_ value measured for compound **2** against ERK5 in our assay is in good agreement with that reported in the literature for compound **1** (Table 2 ▸; Deng *et al.*, 2013 ▸). Our previous results also demonstrated that compound **2**, as an ERK5 tool compound, was able to block downstream ERK5 signalling in a HEK293 cell line which stably co-expressed both a constitutively active variant of MEK5 (MEK5CA) and ERK5 (Gavine *et al.*, 2015 ▸). Compounds **3**–**6** were identified as potential novel ERK5 inhibitors originating from a high-throughput screen of the AstraZeneca corporate collection using the TR-FRET assay. Compounds **4**–**6** are close analogues of one another. It is worthwhile pointing out that the biochemical assay that we employed to measure ERK5 activity uses phosphorylated ERK5 and thus provides a measure of binding to the phosphorylated state *via* inhibition of activity, while the ERK5 crystal system that we employed uses nonphosphorylated ERK5 protein and thus provides a readout of binding to the nonphosphorylated state.

### 3.2. Overall structures of ERK5–inhibitor complexes   

**Figure 2:**
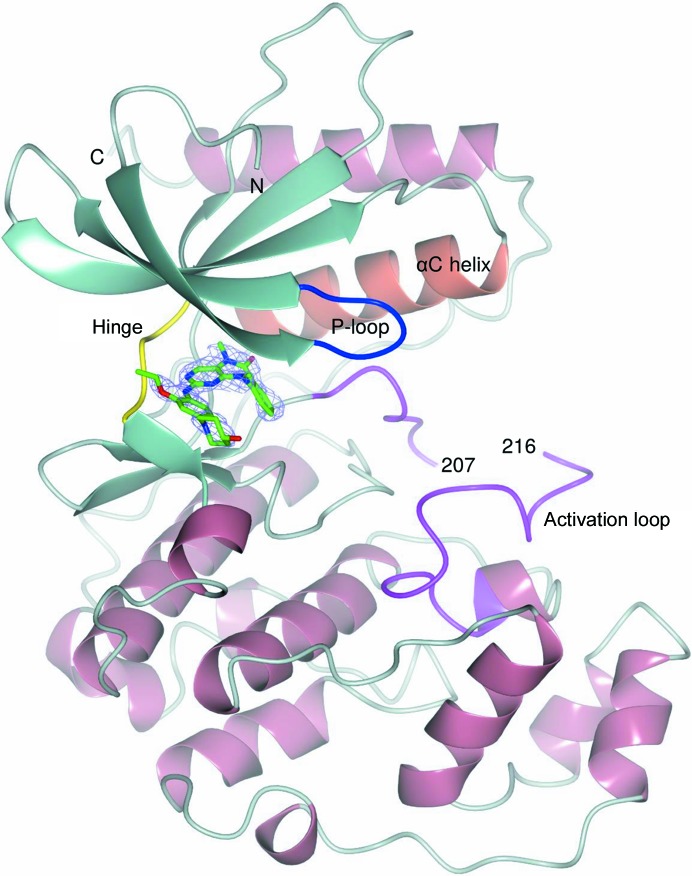
Structure of ERK5 in complex with XMD8-92 (compound **2**). ERK5 is shown in ribbon representation with the hinge region, P-loop, αC helix and activation loop coloured yellow, blue, coral and magenta, respectively. The central portion of the activation loop (amino acids 208–215) is missing from the model. XMD8-92 (compound **2**) binds in the ATP-binding site and is shown as a stick model with C atoms in green. The 2*mF*_o_ − *DF*_c_ OMIT electron density for compound **2** is shown as a lilac mesh contoured at 1.0σ.

The structures of the ERK5 kinase domain in complex with compounds **2**–**6** were solved by molecular replacement to resolutions ranging from 1.65 to 2.79 Å (Table 1 ▸) and contain one molecule of ERK5 in the crystallographic asymmetric unit, which displays the characteristic two-lobe kinase domain structure. Key features of the ERK5 kinase domain are indicated in Fig. 2 ▸, including the hinge region (Leu137–Glu141) which links the two lobes, the glycine-rich P-loop (Ile61–Val69) that encloses the ATP-binding site, the αC helix and the activation loop, which is bounded by the conserved DFG (Asp200–Gly202) and APE (Ala229–Glu231) motifs. The ERK5 C-terminus extends beyond the canonical kinase domain, forming an extended loop followed by an α-helix, which pack against the N-terminal lobe, stabilizing the αC helix in an active-like conformation. Residues Asp53–Arg392 have been modelled in the structures of the complexes with compounds **3**–**6**. Residues 208–215, corresponding to the central portion of the activation loop, could not be modelled in the complex with compound **2**. In a subset of the structures, sufficient electron density was visible at the N- and C-termini to allow up to four additional residues to be modelled. Outside the P-loop, all five structures are very similar, exhibiting an overall root-mean-square deviation (r.m.s.d.) of 0.52–0.78 Å over 323–334 C^α^ coordinates.

**Figure 3:**
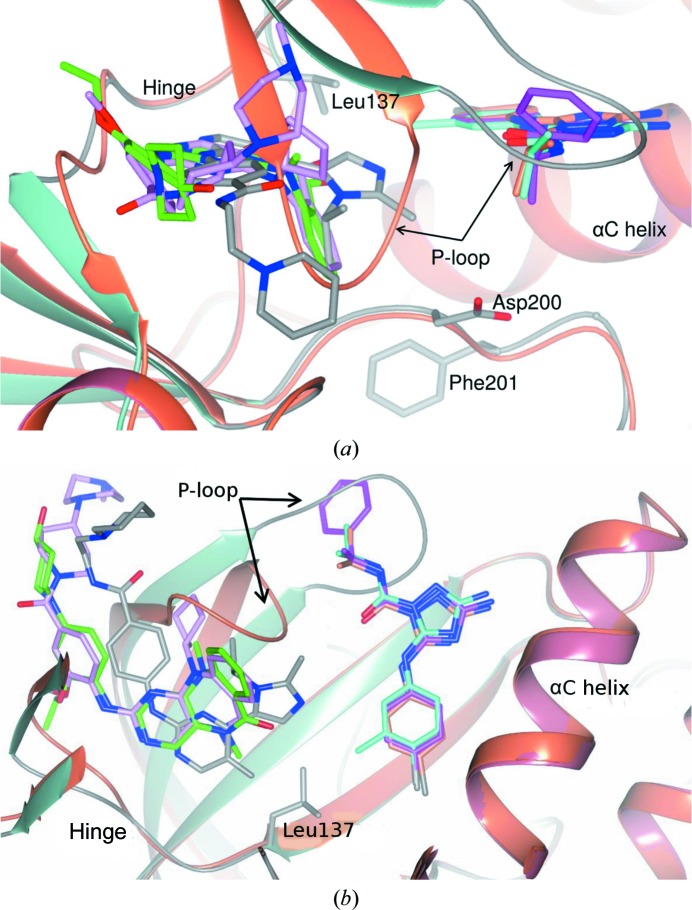
Comparison of the co-crystal structures of ERK5 in complex with the conventional ATP-binding site inhibitors **1** (PDB entry 4b99), **2** and **3** with those of ERK5 in complex with the allosteric inhibitors **4**, **5** and **6**. Displacement of the P-loop into the ATP-binding site upon binding of **4**, **5** and **6** is highlighted. The protein backbone for the complex of ERK5 with compounds **1** (coloured by secondary-structural element with β-sheets, α-­helices and loops in sea green, pale crimson and grey, respectively) and **4** (coral) is shown as a worm representation. Bound ligands are rendered as sticks with C atoms coloured pink (compound **1**), green (compound **2**), grey (compound **3**), coral (compound **4**), cyan (compound **5**) and magenta (compound **6**). The side chains of the gatekeeper residue (Leu137) and the DFG motif (Asp200 and Phe201) from the complex of ERK5 with compound **1** are shown as sticks with C atoms in grey. In (*b*), the view has been rotated ∼90° with respect to (*a*), such that the viewer looks onto the kinase N-lobe, and the C-lobe has been omitted for clarity.

An overlay of the co-crystal structures of ERK5 in complex with the inhibitors **2**–**6** on the published co-crystal structure with compound **1** (PDB entry 4b99) is shown in Fig. 3 ▸. Interestingly, these inhibitors occupy two different binding sites on ERK5. Compounds **2** and **3**, like compound **1**, bind at the conventional ATP-binding site, while compounds **4**, **5** and **6** bind at a novel allosteric binding site. The allosteric binding site is located in a cleft between the P-loop and the αC helix adjacent to the ATP-binding site. This type of allosteric binding site has been reported for other kinases (Chaikuad *et al.*, 2014 ▸; Han *et al.*, 2014 ▸; Ohren *et al.*, 2004 ▸; Martin *et al.*, 2012 ▸); however, this is the first time it has been described in ERK5. Among these three allosteric inhibitors, **5** is the most potent, with an IC_50_ of 2.3 µ*M* in the biochemical assay (Table 2 ▸).

**Figure 4:**
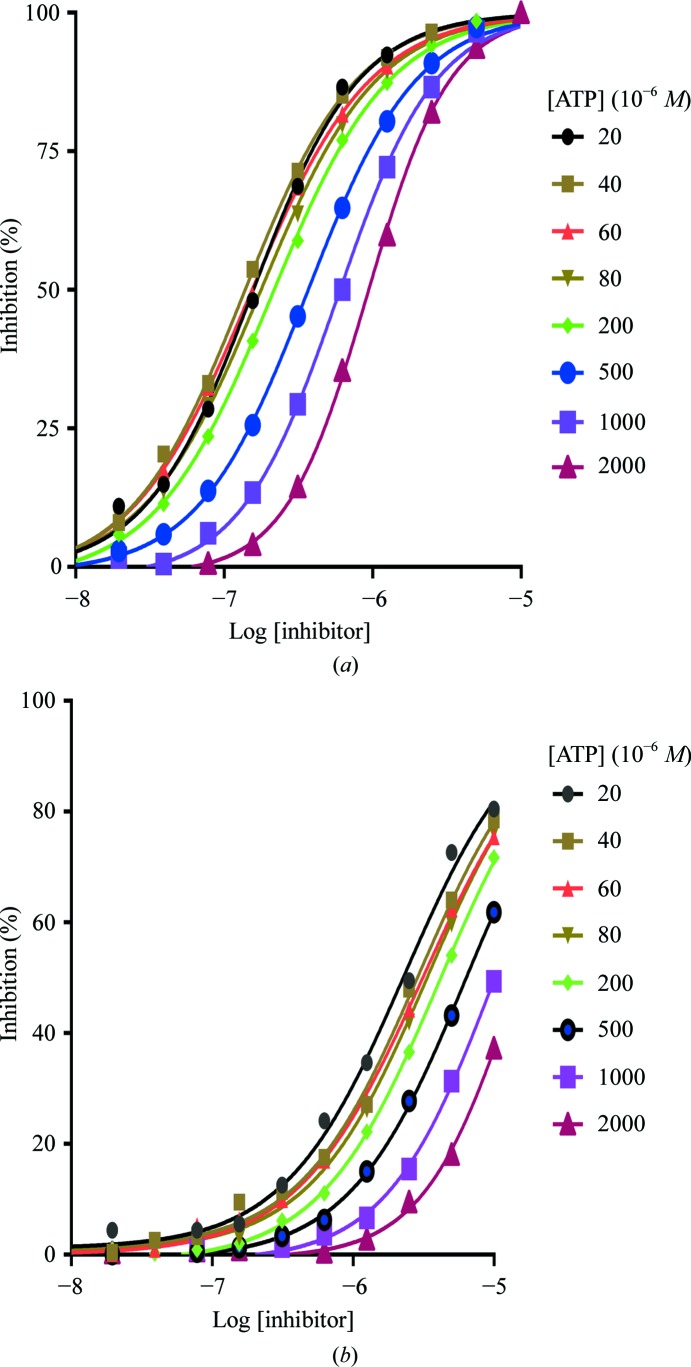
Influence of ATP concentration on IC_50_ curves for (*a*) compound **2** and (*b*) compound **5**.

It can be seen from Fig. 3 ▸ that, as a consequence of forming the allosteric binding site, the P-loop is pushed away from the αC helix to block the ATP-binding site, thus suggesting that these allosteric inhibitors should be ATP-competitive. An ATP-competition study was therefore carried out to verify this hypothesis. A comparison of the IC_50_ curves at different ATP concentrations for the allosteric inhibitor **5** and the reference compound **2** shows that with increasing ATP concentration the IC_50_ values for both compounds **2** and **5** consistently increase (Table 3 ▸ and Fig. 4 ▸). These results suggest that the ERK5 inhibition of compound **5**, like that of compound **2**, is indeed ATP-competitive.

### 3.3. Binding modes of compounds **2** and **3**, two conventional ERK5 inhibitors   

**Figure 5:**
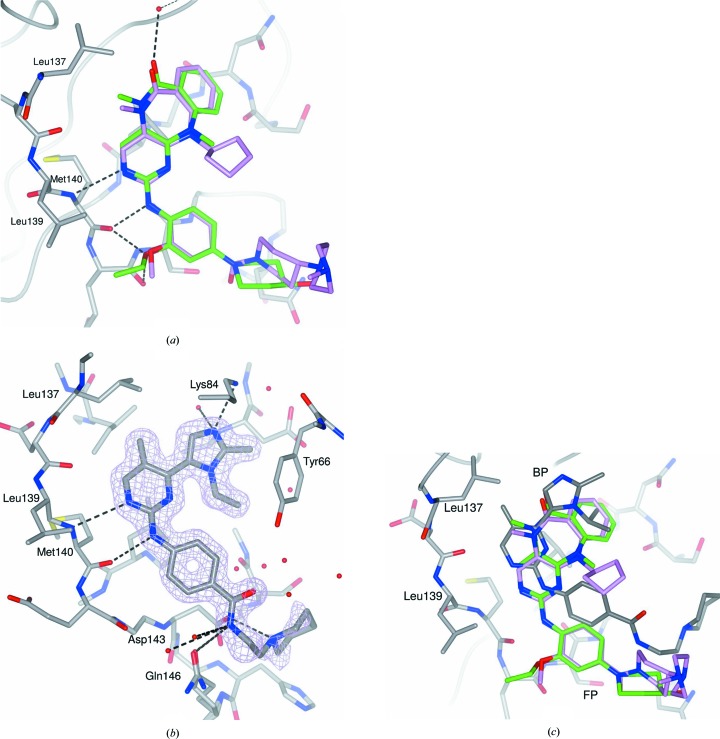
(*a*) An overlay of the structures of ERK5 in complex with compound **1** (C atoms in pink) and compound **2** (C atoms in green) highlights their similarity in binding mode. Hydrogen bonds between compound **2** and ERK5 are shown as black dashed lines. (*b*) Compound **3** binds to the ATP-binding site of ERK5, making hydrogen-bond interactions with the hinge (Met140), solvent channel (Asp143 and Gln146) and catalytic lysine (Lys84). The 2*mF*_o_ − *DF*_c_ OMIT electron density for compound **3** is shown as a lilac mesh contoured at 1.5σ. Hydrogen bonds are shown as dashed black lines. ERK5 and compound **3** are shown in stick representation with C atoms in grey. (*c*) Compounds **1**, **2** and **3** show subtle differences in binding mode as a result of differences in their substitutions, although they utilize a similar aminopyrimidine hinge-binding motif. ERK5 is rendered as sticks with C atoms in grey (complex with compound **1**). Compounds are rendered as sticks with C atoms in pink (compound **1**), green (compound **2**) or grey (compound **3**). Leu137 (the gatekeeper residue), Leu139 in the hinge, the front pocket (FP) and the back pocket (BP) are labelled.

Unsurprisingly, given the similarity of the two compounds, the structure of ERK5 in complex with compound **2** is similar to that of ERK5 in complex with compound **1** (PDB entry 4b99; Fig. 5 ▸ *a*). Compound **2** has a primary alcohol in place of the methylpiperazine group of compound **1**, an *N*-methyl in place of the *N*-cyclopentyl substituent on the tricyclic ring system, and an *O*-ethyl in place of an *O*-methyl at the *meta*-position of the phenyl group. These changes combine to yield a compound with a similar inhibitory potency to compound **1** (Table 2 ▸).

As observed for the reference compounds **1** and **2**, compound **3** binds at the conventional ATP-binding site (Figs. 3 ▸, 5 ▸ *b* and 5 ▸ *c*). The aminopyrimidine motif forms a hydrogen-bond network with the backbone of Met140 in the ERK5 hinge region. The imidazole ring is located in the back pocket (BP; Fig. 5 ▸ *c*) of the ATP-binding site and one aromatic N atom in the ring forms a hydrogen bond to the side-chain amino group of Lys84. The amide substituent on the phenyl ring of compound **3** extends into the solvent-accessible channel (the so-called front pocket; FP) and the amide NH donates a hydrogen bond to the side-chain amide carbonyl O atom of Gln146. The terminal piperidine ring provides an additional ionic interaction with the likely deprotonated carboxylic acid of Asp143.

An overlay of the structures of ERK5 in complex with compounds **1**, **2** and **3** (Fig. 5 ▸ *c*) shows that the aminopyrimidine ring of compound **3** penetrates deeper into the ERK5 binding pocket than the equivalent hinge-binding motifs of compounds **1** and **2**. The tricyclic ring systems of compound **1**, and to a somewhat lesser extent compound **2**, however, occupy a greater volume than the equivalent region of compound **3**, with the benzene-ring fragment extending downwards to fit into the base of the ATP-binding site, whilst in compound **1** the *N*-cyclopentyl substituent points upwards towards the P-loop and packs against Ile61. The *N*-isopropyl group of compound **3** is positioned similarly to the benzene ring in the 6,7,6 ring systems of compounds **1** and **2**. In contrast to the poorly defined conformation of the piperidine ring in compound **3**, the solvent-exposed piperidine–piperazine moiety of compound **1** is directed out of the ATP-binding site and is located adjacent to the P-loop.

### 3.4. Binding mode of allosteric ERK5 inhibitors   

**Figure 6:**
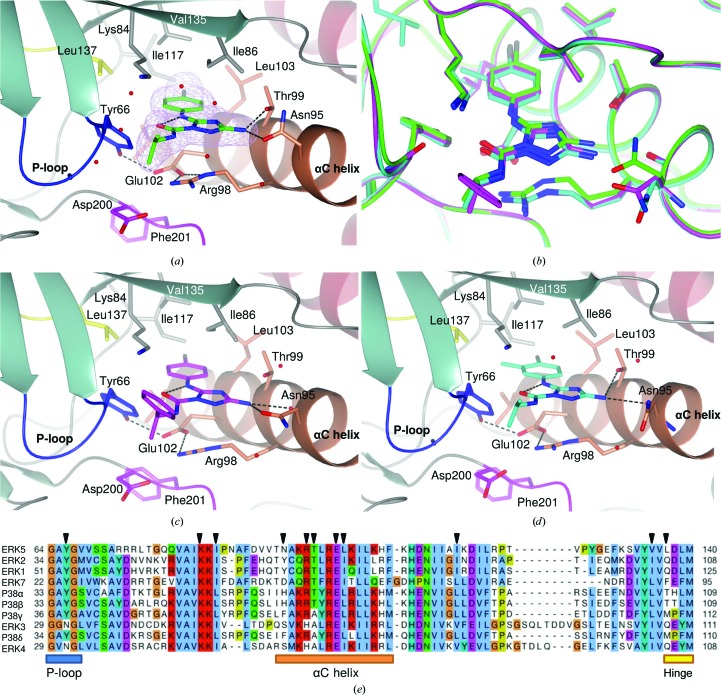
(*a*, *c*, *d*) Compounds **4** (C atoms in green) (*a*), **6** (C atoms in magenta) (*c*) and **5** (C atoms in cyan) (*d*) bind to a novel allosteric pocket situated between the αC helix (coral) and the P-loop (blue) of ERK5. Hydrogen bonds are shown as dashed black lines. ERK5 is rendered in ribbon representation and coloured as in Fig. 2 ▸, with selected side chains, including those of the gatekeeper (Leu137) and the DFG motif (Asp200, Phe201 and Gly202), shown as sticks. In (*a*) the 2*mF*_o_ − *DF*_c_ OMIT electron density for compound **4** is shown as a lilac mesh contoured at 1.0σ. (*b*) Compounds **4**, **5** and **6** show similar binding modes. ERK5 is shown in worm representation with selected side chains and bound compounds rendered as sticks with C atoms in green (complex with compound **4**), cyan (complex with compound **5**) or magenta (complex with compound **6**). (*e*) Sequence alignment for members of the MAPK family, highlighting areas of conservation in the region of the allosteric binding pocket. Residues contacting compound **5** are indicated by filled black triangles above the sequence. Key secondary-structural elements are shown below the sequence.

Compounds **4**, **5** and **6** bind at a different binding site to compounds **1**, **2** and **3**. This site lies adjacent to the ATP-binding site (Figs. 3 ▸ and 6 ▸ and Supporting Information). The *para*-chloro-substituted phenyl ring of **4** protrudes deeply into a hydrophobic pocket which is formed by the side chains of Ile86, Leu103, Ile117 and Val135 from the β3 strand, the αC helix and the β4 and β5 strands, respectively (Fig. 6 ▸ *a*). An intramolecular hydrogen bond between the amide O atom and the aniline N atom forms a pseudo-bicyclic ring system that maintains the compound in a planar conformation. The primary amine donates hydrogen bonds to the side chains of both Asn95 and Thr99 from the αC helix. One face of the pocket is defined by the side chains of Arg98 and Glu102 from the αC helix, which form a favourable salt bridge with one another. π–π stacking is observed between the triazole ring and the guanidinium group of Arg98. Formation of a hydrogen bond between Glu102 and the hydroxyl group of Tyr66 from the tip of the P-loop stabilizes the P-loop in a closed conformation. The terminal isopropyl group of compound **4** is largely solvent-exposed and yet is well defined in the electron density (Fig. 6 ▸ *a* and Supporting Information).

Compounds **5** and **6** are close analogues of compound **4**, and they adopt a similar binding mode (Figs. 6 ▸ *b*, 6 ▸ *c* and 6 ▸ *d* and Supporting Information). The *meta* methyl substituent on the chlorophenyl ring of compound **5** is well accommodated in the binding pocket, and is likely to contribute to the increased potency measured for this inhibitor (Table 2 ▸). The 2-methylphenyl substituent of compound **6**, which replaces the isopropyl group of compounds **4** and **5**, is solvent-exposed and does not appear to contribute any interactions, consistent with the limited potency of compound **6** (Table 2 ▸) and the ambiguity of the electron density for this portion of the molecule (Supplementary Fig. S2).

As observed for the complex of compound **1** with ERK5 (Yang *et al.*, 2010 ▸), compounds **2**–**5** bind to a ‘DFG-in’ conformation of ERK5 in which both the hydrophobic R-spine (residues Leu106, Ile117, His180 and Phe201 in ERK5) and C-spine (Val69, Ala82, Leu144, Leu188, Leu189, Val190, Ile251 and Met255) are intact. Compared with the structures of ERK5 in complex with the hinge-binding compounds **1**–**3**, however, the P-loop is pushed away from the αC helix towards the ATP-binding site upon allosteric inhibitor binding (Fig. 3 ▸; compare the grey and coral P-loops). Differential flexibility in the P-loop amongst kinases has been implicated in the selectivity of certain conventional ATP-binding site-targeted kinase inhibitors (Guimarães *et al.*, 2011 ▸), and may have implications for the selectivity of our allosteric ERK5 inhibitors (see §[Sec sec3.5]3.5). In contrast to the P-loop, the αC helix orientation is unchanged upon the binding of compounds **4**–**6** (Fig. 3 ▸). The position of this helix is constrained in ERK5 by packing against the adjacent C-terminal extension (Pro361–His389; Fig. 2 ▸). Although the αC helix remains in an active-like conformation, the salt bridge between Lys84 (from strand β3) and Glu102 (from the αC helix), which is characteristic of an active kinase, is disrupted (Fig. 6 ▸ *a*). The side chain of Lys84 is displaced by binding of compounds **4**–**6** and reorients to form a hydrogen bond to the backbone carbonyl of Val68 from the P-loop. Glu102, meanwhile, adopts an alternative rotamer allowing the formation of a salt bridge with Arg98 and a hydrogen bond to Tyr66. This new network of hydrogen bonds contributes to stabilization of the allosteric pocket. Conservation of the ERK5 αC helix conformation upon binding of compounds **4**–**6** further differentiates this inhibitor-binding mode from those of other reported allosteric kinase inhibitors (see §[Sec sec3.6]3.6 and Fig. 9).

### 3.5. Allosteric and conventional ERK5 inhibitors show differing selectivity profiles   

**Figure 7:**
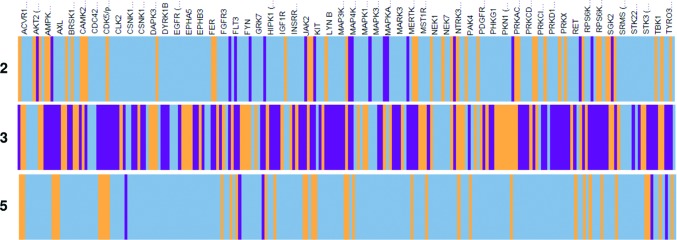
Kinase-panel selectivity for ERK5 inhibitors **2**, **3** and **5**. The violet lines indicate kinases for which the percentage inhibition is above 80%, the orange lines those where it is between 40 and 80%, and the light blue lines those where it is less than 40%.

Compounds **2**, **3** and **5** were tested against a panel of 225 kinases representative of the kinome to assess the selectivity of these three different scaffolds. The kinase selectivity-profile data (Fig. 7 ▸ and Supporting information) show that compound **5** is the most selective, while compound **3** is the most promiscuous, with the selectivity of compound **2** being intermediate. The selectivity of compound **1**, and related compounds such as compound **2**, for ERK5 over other kinases has been extensively discussed (Elkins *et al.*, 2013 ▸).

Consideration of the key interactions made between compound **5** and ERK5 explains the selectivity of this compound for ERK5 over other members of the MAPK family, and suggests that compounds **4** and **6** will show similar selectivity (Fig. 6 ▸ *e*). The side chain of Thr99 forms a hydrogen bond to the amino group of compound **5** (Fig. 6 ▸ *a*); this is one of only two hydrogen bonds between ERK5 and this compound. The residue equivalent to Thr99 is conserved as a threonine in ERK1, ERK2, p38α and p38β, but is an alanine in ERK3, ERK4, p38γ and p38δ. A second nonconserved residue in the allo­steric binding site is Val135, which forms one face of the pocket, and against which the phenyl moiety of the allosteric binders packs. In ERK1–4, ERK7/8 and the p38 MAPKs, this residue is a bulkier isoleucine or leucine. Similarly, Ile86, which also contributes to this face of the pocket, although conserved across the ERK MAPKs, is a leucine in the p38 MAPKs. The so-called gatekeeper residue Leu137 lies within 3.5 Å of the methyl substituent on the phenyl ring of compound **5**. The identity of the gatekeeper exhibits considerable variability amongst members of the MAPK family and is a glutamine in ERK1, ERK2, ERK3 and ERK4, a threonine in p38α and p38β, a methionine in p38γ and p38δ and a phenylalanine in ERK7/8, thus significantly altering the properties of this part of the allosteric pocket. Three additional hydrophobic residues (Leu103, Leu106 and Ile117) which contribute to the binding pocket for compound **5** show variation across the MAPK family (Fig. 6 ▸ *e*). Consistent with the differences in amino-acid composition of the allosteric binding pocket, compound **5** showed less than 10% inhibition when tested against a panel of MAP kinases including ERK1, ERK2 and p38 (Supporting Information).

Compound **5** shows greater than 80% inhibition against five other kinases in the panel of 225: colony-stimulating factor 1 receptor (CSF1R) kinase, FLT4 [vascular endothelial growth factor receptor (VEGFR) 3 kinase], glycogen synthase kinase (GSK) 3α, spleen tyrosine kinase (SYK) and nonreceptor tyrosine protein kinase TYK2 (Fig. 7 ▸ and Supporting Information). The reason for this cross-reactivity is not clear from sequence alignments or the available structures for these five kinases and may arise from an alternative binding mode in which compound **5** binds at the ATP-binding site through a canonical hinge-binding donor–acceptor–donor motif.

### 3.6. ERK5 allosteric inhibitors in the context of the kinome   

Kinase inhibitors typically fall into two major classes (Knight & Shokat, 2005 ▸): type I inhibitors that target a kinase active state and bind at the ATP-binding site (such as compounds **1**, **2** and **3**) and type II inhibitors that target an inactive form. The latter are usually characterized by a ‘flipped-out’ conformation of the activation-loop DFG motif and extend beyond the ATP-binding site to additionally occupy a hydrophobic pocket (Zhang *et al.*, 2009 ▸). Selectivity can be major challenge in both cases (Müller *et al.*, 2015 ▸). Allosteric inhibitors such as **4**, **5** and **6** are much rarer.

**Figure 8:**
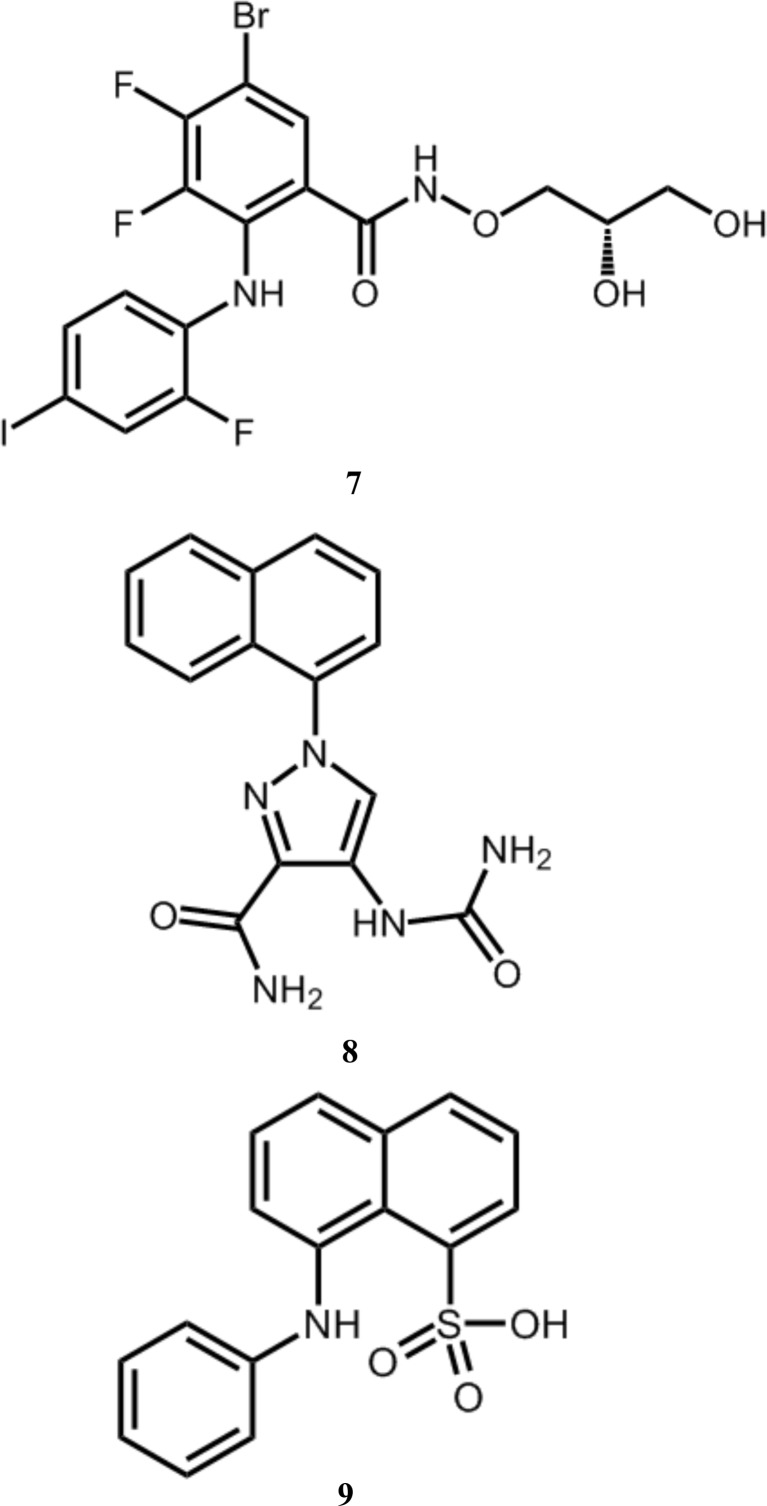
Chemical structures of representative allosteric inhibitors of MEK, ITK and CDK2.

**Figure 9:**
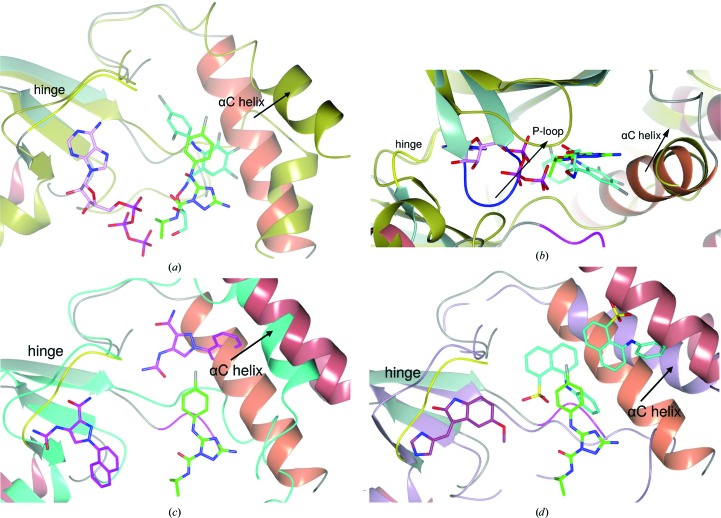
Comparison of the ERK5 allosteric ligand-binding mode with allosteric inhibitors of (*a*, *b*) MEK1/2 (PDB entries 1s9i and 1s9j; Ohren *et al.*, 2004 ▸), (*c*) ITK (PDB entry 4m0y; Han *et al.*, 2014 ▸) and (*d*) CDK2 (PDB entry 3py1; Betzi *et al.*, 2011 ▸) highlights the changes in αC helix orientation and P-loop conformation associated with inhibitor binding. In (*a*), (*c*) and (*d*) the structures are oriented such that the viewer looks onto the kinase C-lobe, and the N-terminal β-sheet has been omitted for clarity. In (*b*) the view has been rotated 90° with respect to (*a*) to show the differences in P-loop conformation. Structures were superposed by matching residues corresponding to the β-sheet and hinge region of the N-terminal lobe of ERK5 kinase (amino acids 55–61, 70–88, 108–123 and 131–140). ERK5 (coloured as in Fig. 2 ▸), MEK (gold), ITK (cyan) and CDK2 (lilac) are shown in ribbon representation. Bound ligands are rendered as sticks with C atoms coloured green (compound **4**), pink (ATP), cyan (MEK inhibitor; compound **7**), magenta (ITK inhibitor; compound **8**), purple (SU9516) and cyan (ANS; compound **9**).

The most thoroughly characterized allosteric kinase inhibitors are those targeting MEK1/2; for example, PD318088 (compound **7**; Fig. 8 ▸), cobimetinib and selumetinib (Ohren *et al.*, 2004 ▸; Hatzivassiliou *et al.*, 2013 ▸; Templeton & Musib, 2015 ▸). These compounds bind in a similar region of the MEK kinase as compounds **4**, **5** and **6** in ERK5, and likewise bury a *para*-halogen-substituted phenyl ring within a broadly equivalent pocket (Figs. 9 ▸ *a* and 9 ▸ *b* and Supporting Information). Few specific interactions are conserved, however, and most notably the MEK inhibitors do not reorder the P-loop towards the ATP-binding site (Fig. 9*b* ▸) and are noncompetitive with ATP (Fig. 9 ▸ *b*). Indeed, the MEK inhibitors can directly interact with the ATP-coordinated Mg^2+^ ion, forming a MEK–ATP–Mg^2+^–inhibitor complex.

More recently, allosteric inhibitors of ITK (Han *et al.*, 2014 ▸) have been reported (compound **8**; Fig. 8 ▸). Again, these inhibitors bind in a similar region of the kinase fold; however, the pocket is more deeply buried compared with the site identified here for ERK5 (Fig. 9 ▸ *c* and Supporting Information). The specific interactions also differ markedly; in the ITK case a buried network of hydrogen bonds is formed, coordinating both a urea and an amide group within the allosteric pocket, whilst a naphthyl group distorts the αC helix. As for the MEK allosteric inhibitors, the ITK allosteric inhibitors differ from the ERK5 allosteric inhibitors presented here in that they are noncompetitive with ATP.

A third example of allosteric inhibitors binding in this region of the kinase fold is provided by a series of naphthalene sulfonates binding to CDK2 (Martin *et al.*, 2012 ▸; ANS; compound **9**; Fig. 8 ▸). The binding mode of these inhibitors more closely resembles that of the ITK allosteric inhibitors than the ERK5 ligands described here, with a sulfonate buried analogously to the urea group (Fig. 9 ▸ *d* and Supporting Information). Uniquely in the case of CDK2, a second inhibitor molecule binds at an adjacent allosteric site, displacing the αC helix such that the complex can no longer bind cyclin. Again, these allosteric inhibitors of CDK2 are noncompetitive with ATP.

In both the MEK and ITK allosteric inhibitor examples, broad kinase activity screening has demonstrated that these allosteric ligands are highly selective for their respective targets. Compound **5**, the most potent of the allosteric ERK5 inhibitors identified in this study, also shows a high level of selectivity over other kinases (Fig. 7 ▸ and Supporting Information), supporting the hypothesis that kinase inhibitors that bind at such sites may have advantages over the type I and type II inhibitor classes (Müller *et al.*, 2015 ▸).

## 4. Conclusions   

The X-ray crystal structures of five ERK5 inhibitors bound to the kinase domain of ERK5 revealed two different binding modes. The conventional, so-called type I, kinase inhibitor-binding mode is demonstrated by structures of ERK5 co-crystallized with the aminopyrimidine-containing compounds **2** and **3**, while a novel allosteric binding mode was observed for three triazole-containing compounds (compounds **4**–**6**). The most potent allosteric inhibitor, compound **5**, has an IC_50_ of 2.3 µ*M* in an assay of ERK5 kinase activity, compared with an IC_50_ of 42 n*M* for the conventional kinase inhibitor **3**. The allosteric binding site is a cryptic site that is absent from other structures of ERK5 and is located between the P-loop and the αC helix. As a consequence of the binding of compounds **4**–**6**, the P-loop is pushed away from the αC helix towards the ATP-binding site, suggesting that these allosteric inhibitors will also be ATP-competitive. An ATP-competition study confirmed this hypothesis. Kinase-selectivity screening showed compound **5** to be more selective than the type I inhibitors **2** and **3**, with significant activity against only five kinases out of the 225 tested. This novel ERK5 allosteric binding mode may provide a new route to the design of selective ERK5 inhibitors as therapeutic agents.

## Supplementary Material

PDB reference: ERK5, complex with compound **2**, 5byy

PDB reference: complex with compound **3**, 5byz

PDB reference: complex with compound **4**, 4zsg

PDB reference: complex with compound **5**, 4zsj

PDB reference: complex with compound **6**, 4zsl

Supporting Information including reagent list, protein sequence and figures.. DOI: 10.1107/S2059798316004502/cb5088sup1.pdf

Kinase selectivity data table.. DOI: 10.1107/S2059798316004502/cb5088sup2.xlsx

## Figures and Tables

**Table 1:** Crystallographic statistics Values in parentheses are for the outer resolution shell.

Compound	**2**	**3**	**4**	**5**	**6**
PDB code	5byy	5byz	4zsg	4zsj	4zsl
Beamline	X06SA, SLS	X06SA, SLS	ID23.1, ESRF	I02, DLS	MX2, AS
Wavelength (Å)	1.00003	1.00003	0.972	0.979	0.950
Detector	Pilatus 6M	Pilatus 6M	ADSC Q315 CCD	ADSC Q315 CCD	ADSC Q315 CCD
Data-collection date	11/06/2012	10/05/2012	22/11/2012	11/07/2013	29/08/2013
Space group	*P*4_1_2_1_2	*P*4_1_2_1_2	*P*4_1_2_1_2	*P*4_1_2_1_2	*P*4_1_2_1_2
Unit-cell parameters					
*a* = *b* (Å)	92.79	93.40	92.53	93.04	92.69
*c* (Å)	107.30	116.28	107.48	110.61	111.04
Resolution range (Å)	70.19–2.79 (3.04–2.79)	72.82–1.65 (1.74–1.65)	50.0–1.79 (1.85–1.79)	71.2–2.48 (2.54–2.48)	47.63–2.25 (2.32–2.25)
Completeness (%)	99.9 (100)	99.5 (99.8)	99.4 (98.7)	99.9 (99.8)	99.8 (98.1)
Unique reflections	12125	62054	44410	17842	23699
Multiplicity	5.9 (5.7)	7.1 (7.0)	10.6 (10.8)	10.8 (11.3)	7.9 (7.7)
Mean *I*/σ(*I*)	21.7 (4.0)	24.2 (3.3)	20.9 (1.7)	19.1 (3.5)	17.5 (1.9)
*R*_merge_ †	0.069 (0.440)	0.046 (0.690)	0.055 (1.257)	0.110 (0.719)	0.089 (1.203)
*R* value‡ (%)	22.3	17.4	20.6	17.0	17.7
*R*_free_ § (%)	27.3	19.2	23.5	23.0	21.9
Non-H protein atoms	2739	2960	2794	2794	2737
Non-H ligand atoms	35	34	20	21	25
Solvent molecules	17	337	344	261	276
R.m.s. deviations from ideal values					
Bond lengths (Å)	0.008	0.009	0.010	0.010	0.010
Bond angles (°)	1.07	1.20	1.02	1.06	1.00
Average *B* value for protein (Å^2^)	23.3	21.7	55.2	47.9	46.6
Average *B* value for ligand (Å^2^)	62.0	23.9	39.2	68.2	73.7
Average *B* value for water (Å^2^)	14.5	30.3	57.6	56.6	56.4
φ, ψ angle distribution for residues¶					
In favoured regions (%)	95	95.7	94.3	95.3	95.9
In allowed regions (%)	4.2	3.6	4.8	4.1	3.8
In outlier regions (%)	0.8	0.7	0.9	0.6	0.3

† *R*_merge_ = 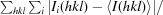 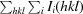.

‡ *R* = 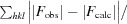 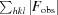.

§ *R*_free_ is the cross-validation *R* factor computed for a test set consisting of 5% of the unique reflections.

¶ Ramachandran statistics as defined by *Coot*.

**Table 2:** Enzymatic potency data for ERK5 inhibitors

Compound	ERK5 IC_50_ (µ*M*)
**1**	0.082†
**2** ‡	0.098
**3**	0.042
**4**	>10
**5**	2.3
**6**	>10

† Data retrieved from the literature (Deng *et al.*, 2013 ▸).

‡ XMD8-92 in the literature (Yang *et al.*, 2010 ▸).

**Table 3:** The derived IC_50_ values at varying ATP concentrations

	[ATP] (µ*M*)							
IC_50_ (µ*M*)	20	40	60	80	200	500	1000	2000
Compound **2**	0.16	0.14	0.15	0.17	0.21	0.36	0.59	0.9
Compound **5**	2.3	2.9	3.1	3.4	4.1	6.3	9.9	14.8
