# Supplementary material for: Discovery of a novel allosteric inhibitor-binding site in ERK5: comparison with the canonical kinase hinge ATP-binding site
Source: Acta Crystallogr D Struct Biol. 2016 Apr 26;72(Pt 5):682–93. doi: 10.1107/S2059798316004502 (PMC4854315; doi:10.1107/S2059798316004502)
Supplement: Supplementary file 1 [file d-72-00682-sup1.pdf]

Acta Cryst D
STRUCTURAL
BIOLOGY

Volume 72 (2016)

Supporting information for article:

Discovery of a novel allosteric inhibitor-binding site in ERK5: comparison with canonical kinase hinge ATP-binding site

Hingming Chen, Julie Tucker, Xiaotao Wang, Paul R. Gavine, Chris Phillips, Martin A. Augustin, Patrick Schreiner, Stefan Steinbacher, Marian Preston and Derek Ogg

Table S1 List of reagents

Reagent	Company	Catalogue Number
PEG 4000	Sigma	95904
PEG 6000	Fluka	81255
MES	Sigma	69890
Tris Base	Fluka	93349
DTT	Molekula	42579915
sodium formate	Sigma	71539
sodium chloride	ROTH	9265.2
glycerol	Sigma	49767
PBS	AppliChem	A0964,9010
magnesium chloride	Fluka	63064
HEPES	AppliChem	A3714,1000
DMSO	Fluka	41639
2,3-butane-diol	Sigma	300349
XMD8-92 (compound 2)	Wuxi PharmaTech	Custom synthesized
Compound 3	Astrazeneca	In-house synthesized
Compounds 4 to 6	Pharmaron	Custom synthesized
ATP	Sigma	A7699
eIF4E-binding protein 1 peptide	PerkinElmer	TRF0128
$MgCl_2$	Sigma	M1028
EGTA	Beyotime	ST068
Tween 20	Sigma	P9416
EDTA	Invitrogen	AM9262
Eu-anti-phospho-eIF4E-binding protein 1antibody	PerkinElmer	TRF0216

Table S2 Translated sequence for human ERK5(46-402) after rTEV cleavage. Residues arising from the rTEV cleavage site are shown in italics.

*G*D^{46}VTFDVGDEYEIIETIGNGAYGVVSSARRRLTGQQVAIKKIPNAFDVVTNAKRTLRELKIL
KHFKHDNIIAIKDILRPTVPYGEFKSVYVVLDLMESDLHQIIHSSQPLTLEHVRYFLYQLLRGL
KYMHSAQVIHRDLKPSNLLVNENCELKIGDFGMARGLCTSPAEHQYFMTEYVATRWYRAPE
LMLSLHEYTQAIDLWSVGCIFGEMLARRQLFPGKNYVHQLQLIMMVLGTPSPAVIQAVGAER
VRAYIQSLPPRQPVPWETVYPGADRQALSLLGRMLRFEPSARISAAAALRHPFLAKYHDPDD
EPDCAPPFDFAFDREALTRERIKEAIVAEIEDFHARREGIRQQIRFQ^{402}

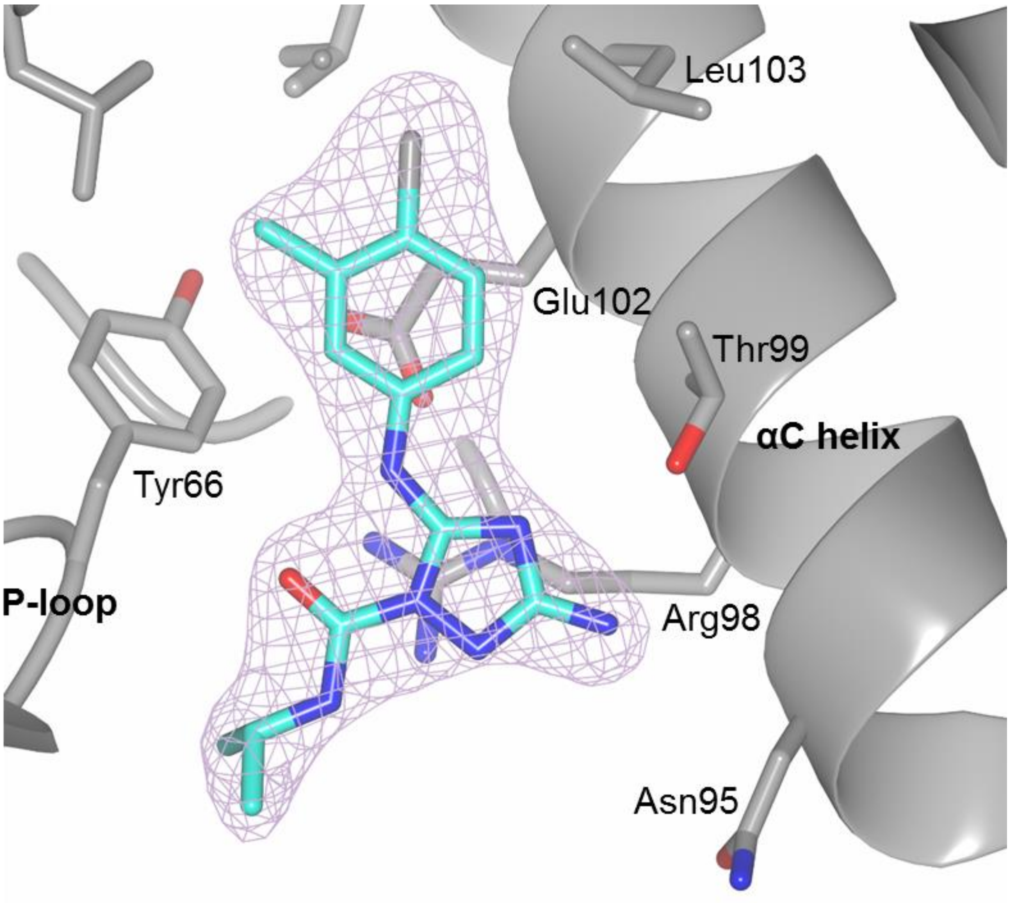

Figure S1 2mF_o-DF_c *OMIT* electron-density map (scaled to 1.0 σ, and represented as a lilac mesh) surrounding compound **5** (carbon atoms in cyan). The compound could be unambiguously fitted in the electron density.

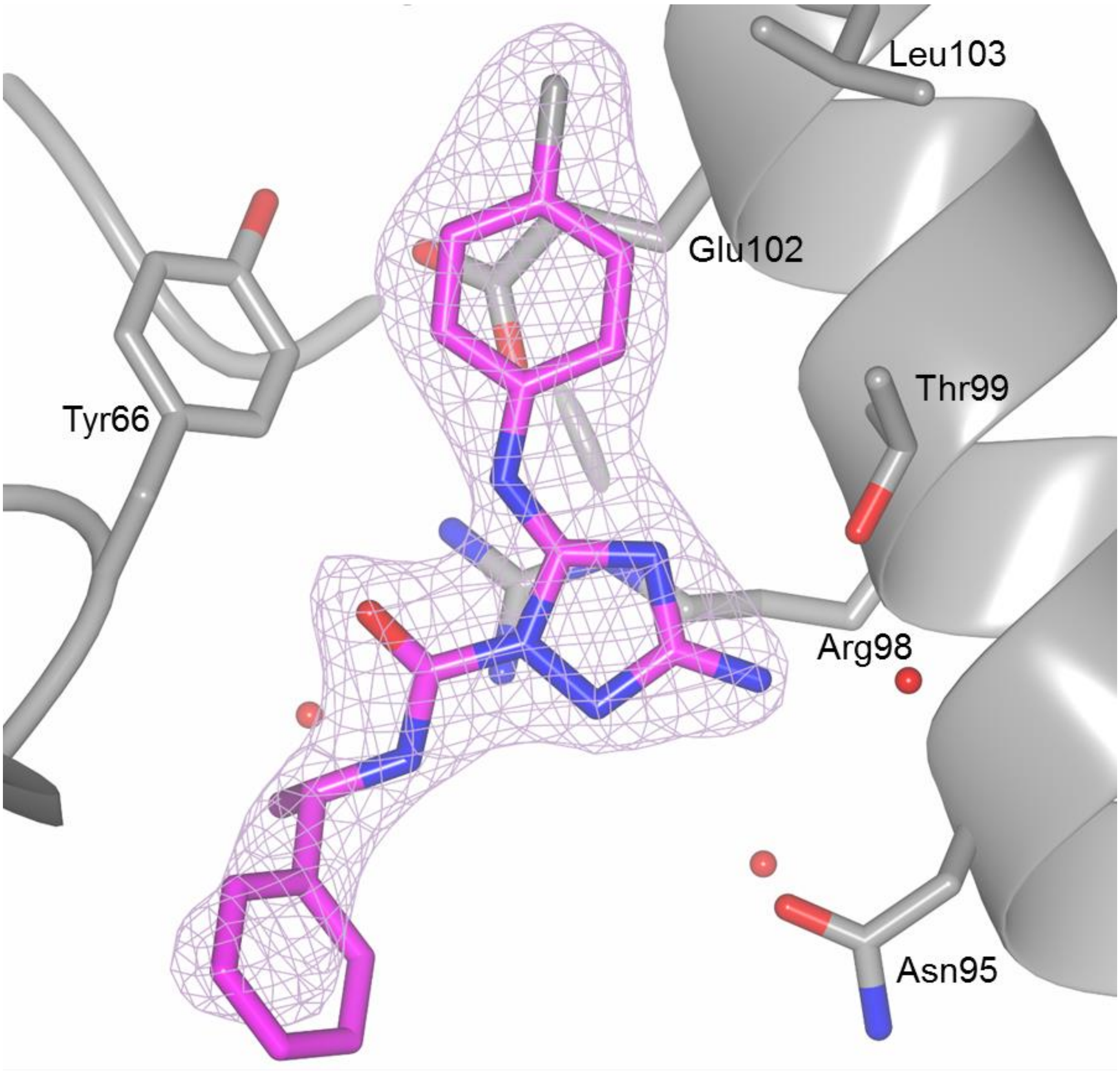

Figure S2 2mF_o-DF_c *OMIT* electron-density map (scaled to 1.0 σ, and represented as a lilac mesh) surrounding compound **6** (carbon atoms in magenta). The compound could be unambiguously fitted in the electron density.

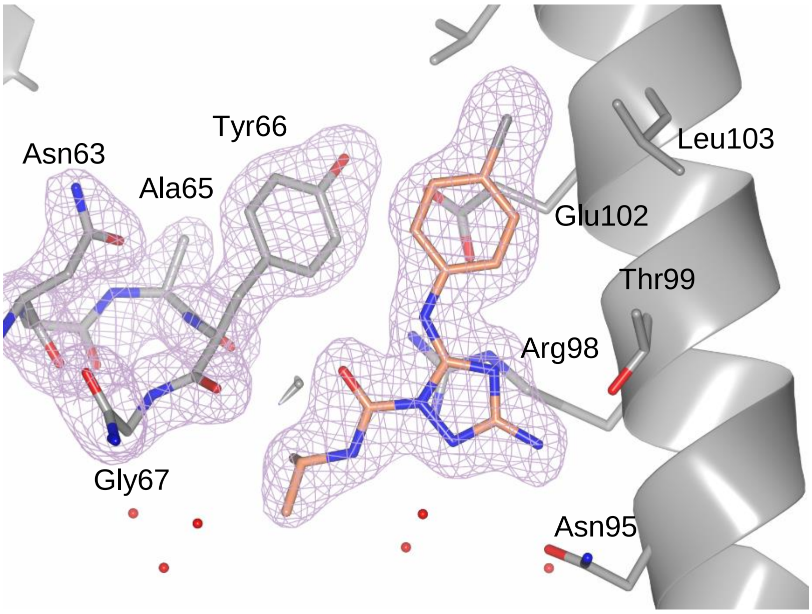

Figure S3 2mF_o-DF_c *OMIT* electron-density map (scaled to 1.0 σ, and represented as a lilac mesh) surrounding the P-loop (Asn63, Gly64, Ala65, Tyr66, Gly67 and Val68, carbon atoms in grey) and compound **4** (carbon atoms in coral). The P-loop residues and the compound could be unambiguously fitted in the electron density.

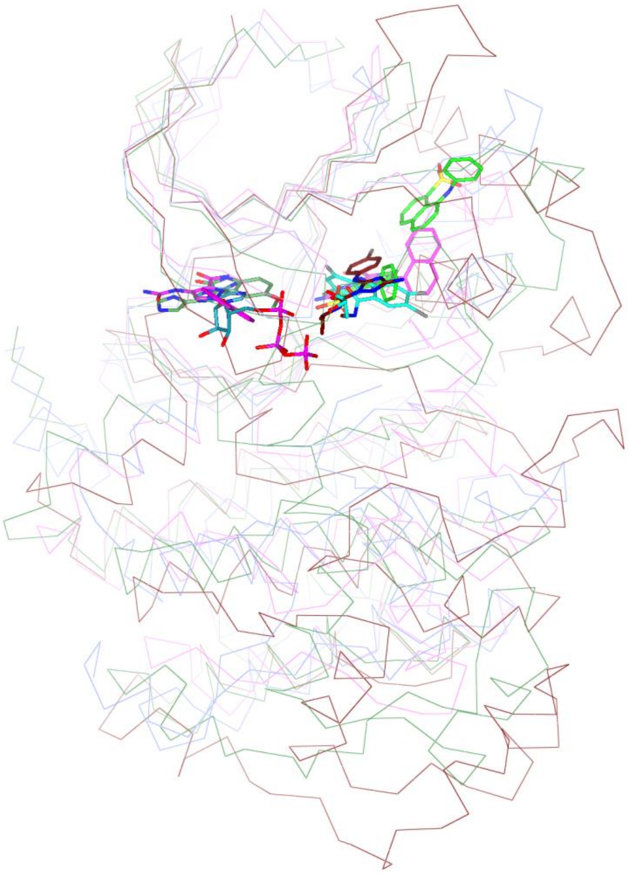

Figure S4 Overlay of the structures of ERK5 in complex with compound **4**, MEK in complex with MgATP and PD318088 (PDB 1S9J), ITK in complex with an allosteric inhibitor (PDB 4M0Y), and CDK2 in complex with SU9516 and ANS (PDB 3PY1). Proteins are rendered as backbone Cα traces in dark brown (ERK5), pale blue (MEK), pink (ITK), dark green (CDK2). Compounds are shown as sticks with carbon atoms coloured tan (compound **4**), dark blue (ATP), cyan (MEK inhibitor, PD318088), magenta (pyrazole carboxamide inhibitor of ITK), dark green (SU9516, a type I inhibitor of CDK2) and green (ANS). Structures were superposed by matching residues corresponding to the β-sheet and hinge region of the N-terminal lobe of ERK5 kinase (amino acids 55-61, 70-88, 108-123, and 131-140).

S1. Z′-LYTE® Assay Protocol

Assay protocol details can be found at:
https://www.thermofisher.com/content/dam/LifeTech/migration/files/drug-discovery/pdfs.par.60256.file.dat/20130430%20ssbk%20customer%20protocol%20and%20assay%20conditions.pdf
